# Supplementary material for: Thermography in ergonomic assessment: a study of wood processing industry workers
Source: PeerJ. 2022 Sep 20;10:e13973. doi: 10.7717/peerj.13973 (PMC9504449; doi:10.7717/peerj.13973)
Supplement: Supplemental Information 2 [file peerj-10-13973-s002.pdf]

One Way Analysis of Variance Tuesday, May 10, 2022, 15:28:11

Data source: Thermography data on laptop

Normality Test (Shapiro-Wilk) Passed (P = 0.469)

Equal Variance Test: Passed (P = 0.968)

Group Name	N	Missing	Mean	Std Dev	SEM
Lumbar - D1	9	0	32.233	0.882	0.294
Lumbar - D2	9	0	31.989	0.991	0.330
Scapular - D1	9	0	32.656	1.137	0.379
Scapular - D2	9	0	32.367	0.971	0.324

Source of Variation	DF	SS	MS	F	P
Between Groups	3	2.084	0.695	0.696	0.561
Residual	32	31.951	0.998		
Total	35	34.036			

The differences in the mean values among the treatment groups are not great enough to exclude the possibility that the difference is due to random sampling variability; there is not a statistically significant difference (P = 0.561).

Power of performed test with alpha = 0.050: 0.049

The power of the performed test (0.049) is below the desired power of 0.800.
Less than desired power indicates you are less likely to detect a difference when one actually exists.
Negative results should be interpreted cautiously.
