# Supplementary material for: Thermography in ergonomic assessment: a study of wood processing industry workers
Source: PeerJ. 2022 Sep 20;10:e13973. doi: 10.7717/peerj.13973 (PMC9504449; doi:10.7717/peerj.13973)
Supplement: Supplemental Information 3 [file peerj-10-13973-s003.pdf]

Normality Test (Shapiro-Wilk) Tuesday, May 10, 2022, 15:23:39

Data source: Thermography data on laptop

Lumbar - D1:	W-Statistic = 0.972	P = 0.914	Passed
Lumbar - D2:	W-Statistic = 0.920	P = 0.396	Passed
Scapular - D1:	W-Statistic = 0.981	P = 0.971	Passed
Scapular - D2:	W-Statistic = 0.950	P = 0.688	Passed

A test that fails indicates that the data varies significantly from the pattern expected if the data was drawn from a population with a normal distribution.
A test that passes indicates that the data matches the pattern expected if the data was drawn from a population with a normal distribution.
